# Mitotic spindle asymmetry in rodents and primates: 2D vs. 3D measurement methodologies

**DOI:** 10.3389/fncel.2015.00033

**Published:** 2015-02-09

**Authors:** Delphine Delaunay, Marc C. Robini, Colette Dehay

**Affiliations:** ^1^Stem Cell and Brain Research Institute, Institut National de la Santé et de la Recherche Médicale, U846Bron, France; ^2^Université de Lyon ILyon, France; ^3^CREATIS (CNRS Research Unit UMR5220 and INSERM Research Unit U1044), INSA-LyonVilleurbanne, France

**Keywords:** asymmetric cell division, cerebral cortex, mouse, primate, corticogenesis

## Abstract

Recent data have uncovered that spindle size asymmetry (SSA) is a key component of asymmetric cell division (ACD) in the mouse cerebral cortex (Delaunay et al., 2014). In the present study we show that SSA is independent of spindle orientation and also occurs during cortical progenitor divisions in the ventricular zone (VZ) of the macaque cerebral cortex, pointing to a conserved mechanism in the mammalian lineage. Because SSA magnitude is smaller in cortical precursors than in invertebrate neuroblasts, the unambiguous demonstration of volume differences between the two half spindles is considered to require 3D reconstruction of the mitotic spindle (Delaunay et al., 2014). Although straightforward, the 3D analysis of SSA is time consuming, which is likely to hinder SSA identification and prevent further explorations of SSA related mechanisms in generating ACD. We therefore set out to develop an alternative method for accurately measuring spindle asymmetry. Based on the mathematically demonstrated linear relationship between 2D and 3D analysis, we show that 2D assessment of spindle size in metaphase cells is as accurate and reliable as 3D reconstruction provided a specific procedure is applied. We have examined the experimental accuracy of the two methods by applying them to different sets of *in vivo* and *in vitro* biological data, including mouse and primate cortical precursors. Linear regression analysis demonstrates that the results from 2D and 3D reconstructions are equally powerful. We therefore provide a reliable and efficient technique to measure SSA in mammalian cells.

## Introduction

Asymmetric cell division (ACD)—unequal division producing two daughter cells with distinct fates—generates cell diversity in prokaryotes and eukaryotes. Significant progress in elucidating the key mechanisms underlying ACD has revealed a high degree of conservation between invertebrates and vertebrates (Knoblich, 2010; Li, 2013).

The conserved mechanisms include sibling cell size asymmetry, which refers to physical asymmetry and has been shown to occur in various cell types and species (including *Saccharomyces cerevisiae* cells, *Drosophila* and *C. elegans* neuroblasts and sensory organ precursor cells). The cellular and molecular machinery responsible for sibling cell size asymmetry is complex and not fully understood (reviewed in Roubinet and Cabernard, 2014). One major player in physical ACD in invertebrates is the asymmetry in spindle poles geometry (Kaltschmidt et al., 2000; Betschinger and Knoblich, 2004; Knoblich, 2010). Recently, we have shown that spindle shape asymmetry (SSA) is a highly conserved mechanism that also operates in the mouse developing mammalian cerebral cortex (Delaunay et al., 2014), where it plays a major role in the tight spatiotemporal control of self-renewal and differentiation during corticogenesis. In the present study, we extend these findings to primates by showing that SSA occurs during the division of macaque monkey cortical precursors. We also demonstrate that SSA magnitude is not biased by the orientation of the spindle with respect to its substrate.

ACD in cortical development occurs in the germinal zones including the apical progenitors of the ventricular zone (VZ) and serves to generate differentiating neurons while amplifying the progenitor pool through self-renewal (Haubensak et al., 2004; Miyata et al., 2004; Noctor et al., 2004; Kriegstein et al., 2006). SSA in apical cortical progenitors is characterized by the unequal organization of the two spindle poles which appear asymmetric in size during metaphase and throughout division, leading to the generation of two daughter cells of distinct size and fate (Delaunay et al., 2014).

Although SSA is easily delineated in invertebrates, its amplitude is smaller in cerebral cortex, making it harder to quantify. Here we present two simple methods based on regular confocal stack acquisitions, which allow accurate SSA measurements using 3D volume estimation and 2D surface area calculation. We describe the procedures for both methods and demonstrate theoretically and empirically that they give similar results. These findings allow us to conclude that, compared to the 3D method, 2D measurement is an efficient and preferred methodology for SSA assessment.

## Methods

### Cell culture

Surgical procedures and animal experimentation were in accordance with European requirements 2010/63/UE. The protocol C2EA42-12-11-0402-003 has been approved by the Animal Care and Use Committee CELYNE (C2EA #42). E13.5 to E14.5 of one mouse brains were electroporated *ex-vivo* (3x 50–70 V pulses of 100 ms duration and 100 ms interval) with 0.1.8 to 2.5 μg/μl DNA. Cortex were dissected in HBSS, cell dissociated with trypsin 1X (Invitrogen) and plated at 4.5. 10^4^ cells per 12 mm diameter poly-D-Lysine (Sigma, 40 μg/ml) coated glass cover slips. Cells were maintained in culture for 1–1.5DIV in DMEM/F12 supplemented with B27 (1:50^*e*^, Invitrogen) and N2 (1:100^*e*^, Invitrogen) and fixed with 37°C 2%PFA for 2–5 min.

### Monkey tissue preparation

Fetuses from timed-pregnant cynomolgus monkeys (Macaca fascicularis, gestation period 165 days) were delivered by caesarian section as described elsewhere (Lukaszewicz et al., 2005). All experiments were in compliance with French national and European laws as well as with institutional guidelines concerning animal experimentation. Surgical procedures were in accordance with European requirements 2010/63/UE. The protocol C2EA42-12-11-0402-003 has been reviewed and approved by the Animal Care and Use Committee CELYNE (C2EA #42). Lethally anesthetized primate fetuses (E63–E80) via intraperitoneal injection of Sodium Pentobarbital 60 mg/kg were perfused through the heart with buffered 4% paraformaldehyde (PFA) for 30 min. After cryoprotection in PBS/Sucrose (10 then 20%), brains were embedded in Tissue-Tek. 20 μm-thick parasagittal cryosections were cut, mounted on superfrost glass slides and immunostained.

### Immunocytochemistry and immunohistochemistry

Cultured cover slips were saturated for 1 h in PBS1X/10% goat serum and incubated with the primary antibody overnight or up to 30 h at 4°C: mouse anti-α-tubulin (sigma, 1:500), rabbit anti-pericentrin (Covance, 1:1000). Sections were then washed in PBS, followed by incubation with appropriate goat fluorescence-conjugated secondary antibodies at room temperature for 2 h (Alexa 488 goat anti Mouse (Invitrogen, 1:1000), Alexa Fluor 555 goat anti-rabbit IgG (Invitrogen, 1/1000). Nuclei were stained with DAPI (0.5 μg/ml). Monkey cryosections were air-dried (30 min) and hydrated in Tris-buffered saline (TBS) for 15 min. Heat-mediated antigen retrieval was performed at 95°C for 15 min. Slices were permeabilized with TBS + TritonX (0.5%) and saturated by incubation in TBS + BSA 1% (= TBSb) + normal donkey serum (10%) + 0.5% triton in TBS for 30 min. Primary antibodies were co-incubated 1 day and 2 nights in TBSb + 0.5% triton at 4°C as follows: mouse anti-alpha-tubulin (sigma, 1:200), rabbit anti-pericentrin (Covance, 1:2000), sheep anti-EOMES (R&D 1:800). Secondary antibodies were co-incubated in Dako Diluent (Dako) 1 h at RT, at the following concentrations: Alexa 488 donkey anti mouse (Invitrogen, 1:200), Alexa 555 donkey anti rabbit (Invitrogen, 1:200), Alexa 647 donkey anti sheep (Invitrogen, 1:200). Nuclear staining was performed using Dapi (Invitrogen, D1306, 2 μg/mL in TBS) 10 min at RT. Sections were mounted in Fluoromount G. Specimens were analyzed with a confocal microscope (Leica DM 6000 CS) allowing acquisition of axial image sequences (“z-stacks”) for 3D quantification. Z-stack images were acquired using a Plan-Apochromat 63 × 1.40 NA oil objective. Excitation wavelength was 488 nm and emission was detected using a long pass filter from 505 nm. Image pixel size was 0.045 μm (x and y) and bit depth 0.998, z-step size 0.3–0.5 μm, and pinhole diameter from 100 μm.

### 3D volume quantification

The 3D volume calculation was based on the original serial confocal acquisitions. The “VolumeJ” program was designed by Denis Ressnikoff (SFR Lyon-Est, CNRS UMS3453–INSERM US7, Centre Commun de Quantimétrie) based on the 3D object counter plugin [Fabrice Cordelieres (fabrice.Cordelieres@curie.u-psud.fr); Jonathan Jackson (j.jackson@ion.ucl.ac.uk)] and the 3D volume viewer [Benjamin Schmid (Bene.Schmid@gmail.com)]. The program is available at https://www.labex-cortex.com/en/users/delphine-delaunay and as.txt in “Supplementary Material.”

### 2D area quantification

Serial sections of metaphase cells from dissociated mouse cortical precursors and from *in situ* monkey VZ precursors, were acquired from 0.2 to 0.6 μm intervals from the top to the bottom of the cells (back to back) in order to measure the entire spindle apparatus. Only metaphase cells presenting equal sized centrosomes were taken into consideration. The area of each spindle pole was measured using Image J on maximal stack projections based on the alpha-tubulin staining. The area of each spindle pole was reported as the percentage difference between the two spindle areas. The folded normal distribution and Permutation test “utilFuncs.R” script as well as data test and instructions are available at https://www.labex-cortex.com/en/users/delphine-delaunay and as.txt in “Supplementary Material.”

### 3D vs. 2D comparative analysis

Two independent sets of data were used to compare 2D and 3D measurements. Eighty one mouse dividing apical precursors in dissociated culture were analyzed. Ninety three monkey apical precursors were analyzed *in situ* on parasagittal sections. The regression analyses were implemented using the MATLAB software.

## Results

### 3D SSA quantification

**Figure 1:**
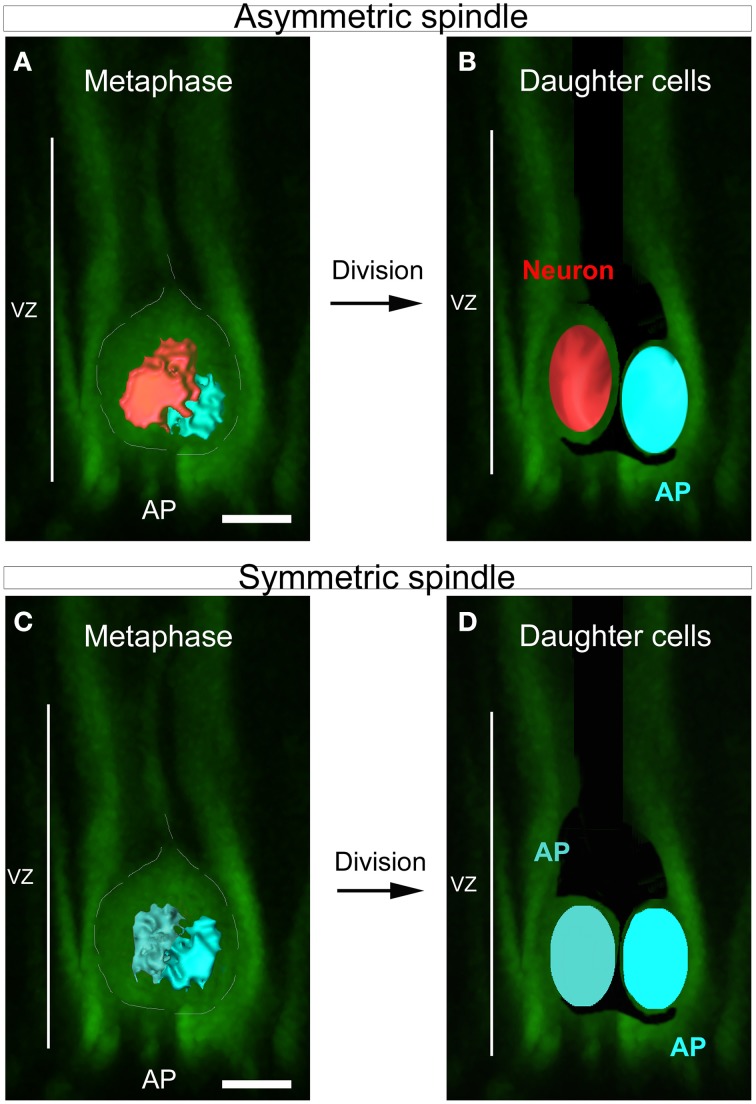
**SSA in the developing cortex. (A–D)** Schematic summarizing the link between spindle shape asymmetry and asymmetric cell division in the cortical ventricular zone. **(A)** Dividing apical progenitor (AP) presenting asymmetric spindle in metaphase. The bigger spindle is highlighted in red and the smaller in blue. The dashed white line indicates the cell shape. **(B)** The cell divides asymmetrically and gives rise to two distinct daughter cells: a neuron (red) and a new dividing AP (blue). The newly born neuron arises from the cell that inherits the bigger spindle. **(C)** Example of symmetric dividing AP displaying spindle of equal sizes at metaphase (dark and light blue). This cell will give rise to daughter cells of equal fate **(D)**. Scale bars: 10 μm.

**Figure 2:**
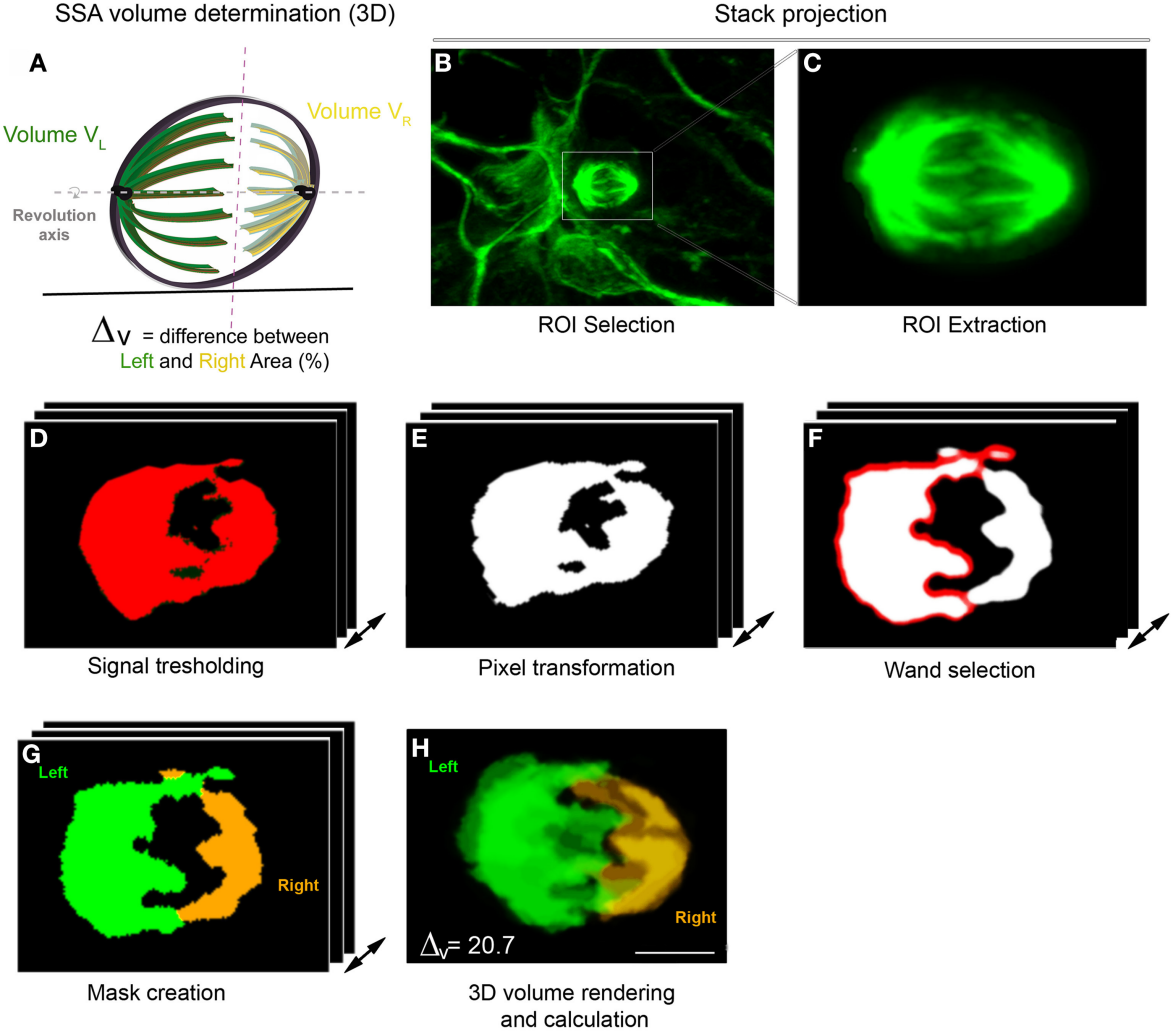
**3D SSA analysis (Volume determination). (A)** 3D representation of the spindle apparatus in metaphase cell. The larger spindle is colored in green and the right spindle in yellow. The intersection between both sides is represented by the red dashed line and both spindle poles rotate along the same revolution axis (gray). **(B–H)** Spindle volume determination using the VolumeJ program. **(B,C)** Stack projection of an *in vitro* metaphase cell stained with α-tubulin. **(C**) ROI extraction of the spindle apparatus. **(D)** Signal tresholding for each optical section. **(E)** Signal transformation in pixels. **(F)** Selection of one spindle pole using the Wand tool. **(G)** Creation of a mask displaying the selected Left and right spindle pole. **(H)** 3D volume rendering and calculation. The volume is independently calculated for each spindle pole. Here, the difference in volume (Δv) is 20.7, typical of an asymmetric cell.

In dividing apical cortical progenitors, spindle size is correlated with daughter cell identity. The daughter cell inheriting the large spindle gives rise to a neuron, and the daughter cell inheriting the small spindle a self-renewing apical progenitor (Delaunay et al., 2014; Figure 1). 3D reconstruction of the two spindle poles, which allows calculation of the volume of each pole, appears as the method of choice for the accurate determination of SSA (Delaunay et al., 2014). Dissociated mouse apical precursors were fixed and stained for α-tubulin, pericentrin and DAPI to reveal the condensed nuclei. For each metaphase cell, optical sections were acquired every 0.2 to 0.5 μm from top to bottom, using confocal microscopy. To avoid problems with tilted or incomplete spindles, we only considered the metaphases displaying centrosomes of equal size (based on the pericentrin staining). We measured each spindle pole volume and named the larger of the two spindles “*Left spindle*” (green, Figure 2A) and the smaller “*Right spindle”* (yellow, Figure 2A). The difference between the left and right spindle poles, called the “3D spindle pole difference,” denoted by Δ_V_ and expressed as a percentage, revealed the SSA magnitude:

$${△}_{\text{V}}\;=\;\left(\frac{V_{\text{L}}-V_{\text{R}}}{V_{\text{L}}+V_{\text{R}}}\;\right)\times 100,$$

where V_L_ and V_R_ denote the volumes of the left and right spindle poles, respectively.

The volume was calculated using a hand designed ImageJ program (VolumeJ, Figure 2; see Methods). The sequential steps of the program are detailed in Table 1. Briefly, the spindle apparatus is extracted from the optical stack (Figures 2B,C) and the signal transformed in pixels after appropriate thresholding (Figures 2D,E). For each optical section, one side of the spindle pole is selected using the wand tool (Figure 2F). The program then considers the non-selected pixels as belonging to the same structure and will create the second spindle pole. A mask appears, displaying one spindle pole in green and the other in yellow. This allows comparison between the mask and the original picture in order to avoid any mistakes. In a final step, the program calculates the spindle volume for each pole based on the extracted voxels (Figure 2H). In the cortex, SSA is consistently maintained in anaphase and throughout mitosis. An example of SSA is shown in Figure 2, where the 3D spindle pole difference, Δ_V_is 20.7, typical of an asymmetric spindle (see Delaunay et al., 2014; Figure 1 Δ_V_ ≤ 10%: symmetric spindle; Δ_V_ > 10%: SSA). Hence, 3D SSA quantification is easy to apply, although its implementation is time consuming. We therefore searched for an alternative, equally reliable method that will allow high scale quantification of SSA and explored the capacity of 2D SSA determination to recapitulate 3D SSA measurements.

**Table 1:** **ImageJ program for 3D spindle reconstruction**.

**The program is downloaded as a stand-alone program and run in ImageJ version 1.47T**
Optical Stacks are taken with a X63 objective, with a minimal pixel resolution of 90 × 90 nm (format 512 × 512, bidirectionnal) and with *z*-value of 0.5 μm.
• Under ImageJ software, open the α-tubulin channel and rename it with a simple name.
• Start the “volume quanti 1_0.ijm” program.
• To avoid background, make a ROI selection close to the spindle, and select “Ok.”
• The macro create two sets of picture: one named “.tif_ROI,” (visualization picture) and the other named “.tif_mask” (3d skeleton).
• On “.tif_mask,” check the correlation between the spindle pixelation and the observed α-tubulin channel (on “.tif_ROI”).
• If it match, click apply to the “threshold” windows.
• Click “ok” on the macro windows to observe simplification of the pixelated shapes.
**Clean non-desired structures recognized as signal (If necessary)**
• Using “.tif_ROI” as models, define the most accurate pixel shape as possible.
• Select non-significant areas on the “.tif_ROI” window, report this selection on “.tif_mask” window and delete it.
• Perform this for all the non-significant areas on each picture of the stack until each one shows only the precise shape of the spindle, as it is observed on the “.tif_ROI” pictures.
• With the “wand” tool, select one side of the spindle and record the selection using “T” button (ROI manager opens automatically). Small fragments can be added to the selection, maintaining “maj” button. Do this for each frame.
• When it's done, select all the ROI at the same time and click “ok” on the macro windows.
• The macro will calculate the volume of selected structures and deduced the volume of the other spindle pole from the non-selected pixels.
• The macro creates two excels files, named.tif_Mask_1 and.tif_Mask_2, giving the volume of each part of the spindle apparatus, plus a colored 3d reconstruction of the spindle (“.tif_color_mask”).

### 2D SSA quantification

**Figure 3:**
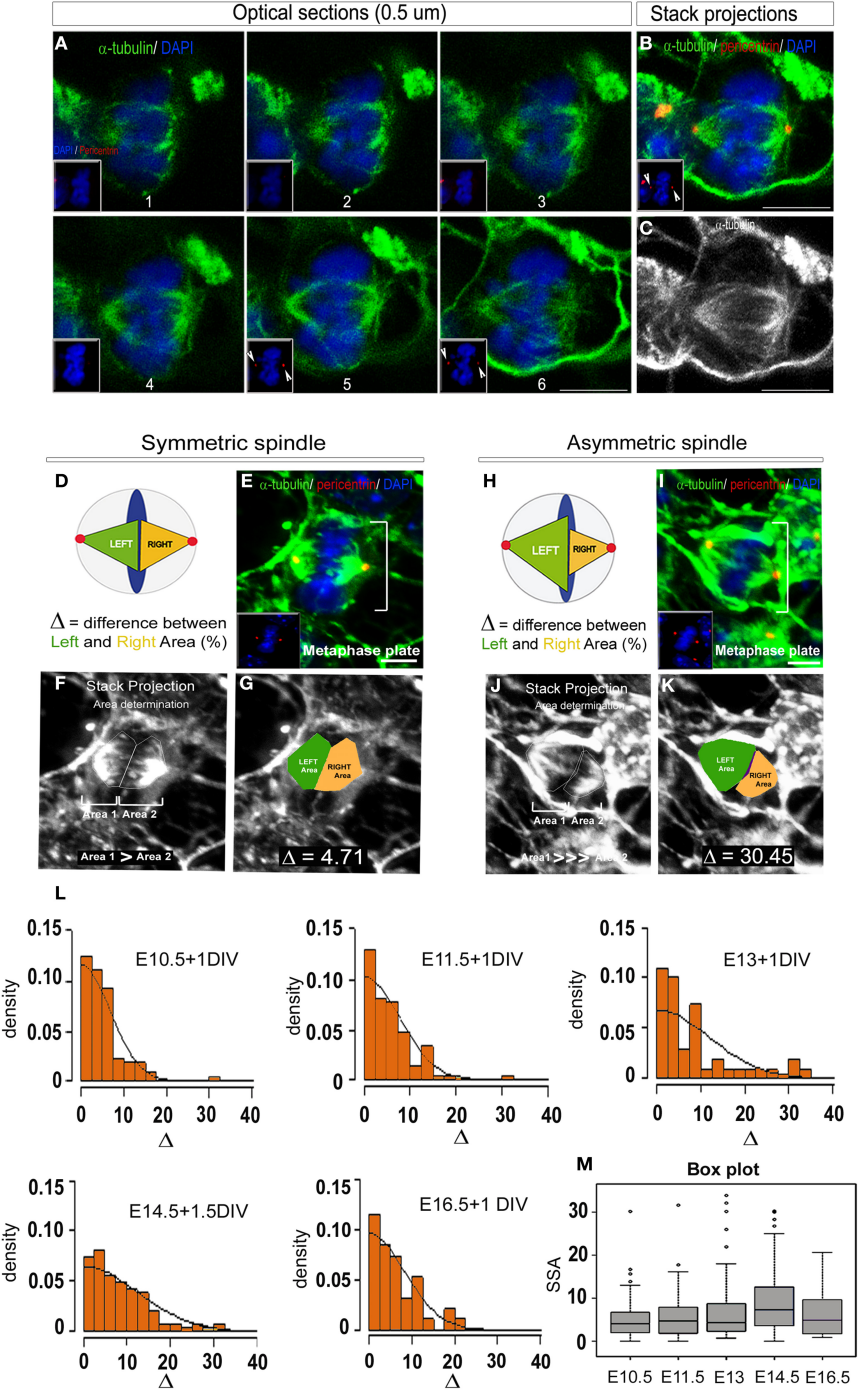
**2D SSA analysis (Area determination). (A)** Optical sections of an E14.5 metaphase cell stained with α-tubulin (to reveal the microtubules, green) and DAPI (blue). The optical sections are taken every 0.5 μm from top to bottom. The magnet sized pictures show the centrosome appearance (red, pericentrin staining). **(B)** Maximum intensity stack projection showing that the entirety of the spindle apparatus is taken into consideration thanks to the equal sized centrosomes (pericentrin, red). **(C)** Maximum intensity stack projection of the same cell revealing the tubules only. **(D–K)** Detailed methods for 2D area determination. **(D,H)** Schematic representation of a symmetric **(D)** and an asymmetric metaphase cells **(H)**. When the spindles are symmetric, each part are of equal sizes (Green = Left spindle, Yellow = Right spindle, arbitrarily consider), conversely, when the spindles are asymmetric, the left spindle area is significantly larger than the right one. **(E,I)** For each cells, a primary reconstruction is made to verify that the centrosome are of equal sizes (**E,I**, yellow dots). **(F,J)** The SSA intensity is determined on maximal intensity stack projection reconstructed under the ImageJ software. Each spindle pole is manually drawn and the corresponding area (1 and 2) calculated. Arbitrarily, the bigger area will be defined as the Left spindle and the smallest as the Right spindle. **(G,K)** The difference between the Left (green) and the Right area (yellow) expressed in percentage will be the unit of measurement, delta (Δ). (**L**) SSA distribution at different time points during *in vitro* cortical cells development. Cells were respectively taken at E10, E11.5, E13 and cultured for 1 day to 1.5 day *in vitro* (DIV). Consistent with previous report (Delaunay et al., 2014), the SSA variation follows a folded normal distribution and parallels the asymmetric cell division kinetics: first an increase with a peak at E14.5 followed by a decrease at E16.5. **(M)** The change in SSA at E14.5 appears highly significant, as demonstrated by the permutation test (*p* = 5.10^−4^). Scale bars: **(A)** 5 μm E, I 10 μm.

To design a reliable 2D SSA quantification method, we analyzed the same data set as for the 3D analysis, that is, dissociated primary cortical precursors from E10.5 to E16.5 for a mean period of 1 day *in vitro* (DIV) (Figure 3). As for the 3D analysis, cells were fixed and stained for α-tubulin, pericentrin and DAPI to reveal the condensed nuclei. For each metaphase cell, optical sections were acquired from top to bottom with 0.2–0.5 μm intervals using a Leica DM6000 confocal microscope. The same criteria as for the 3D quantification were applied (Figures 3A–C). To quantify the SSA, we measured each spindle pole area and named the larger of the two “*Left spindle*” (green, Figures 3D,H) and the smaller “*Right spindle”* (yellow, Figures 3D,H). The difference between the left and the right spindle poles (called the “2D *spindle pole difference”*), denoted by Δ and expressed as a percentage, reveals the SSA magnitude. Using the ImageJ software, for each cell, optical sections were transformed into maximal intensity stack projections (Figures 3E,I). The resulting left and right spindle pole domains were then manually drawn as ROI (Figures 3F, J) and their respective surface area estimated. Let *A*_L_ and *A*_R_, respectively denote the left and right surface areas, the 2D spindle-pole difference is defined by

$$△\;=\;\left(\frac{A_{\text{L}}-A_{\text{R}}}{A_{\text{L}}+A_{\text{R}}}\right)\times 100.$$

Examples of symmetric vs. asymmetric spindle are illustrated Figures 3G,K. In particular, a spindle-pole difference Δ ≥ 20 was often measured in highly asymmetric cells (Figure 3K).

To analyze SSA evolution during cortical development, the spindle-pole difference was evaluated at five distinct developmental stages between E10 and E18. 322 metaphases cells were analyzed and the SSA variations reported using a *folded normal distribution* (see Methods, “2D area quantification”). The *folded normal distribution* represents the distribution of the absolute value of a given variable [the probability measure of the normal distribution on (−∞,0) is folded over to (0,∞)]. The probability density function is reported for each developmental stage and reflects SSA magnitude. This task was performed using an R script specifically designed in our laboratory and freely available (see Methods for details). From E10.5 to E16.5, spindle-pole difference was found to follow the neurogenesis kinetics, with the mean and standard deviation increasing up to E14.5 (neurogenesis) and then decreasing (as illustrated in Figure 3L). The significance values were confirmed using a permutation test. Under the permutation hypothesis, it is assumed that Δ is distributed evenly across ages, so that randomly permuting the labels of the ages across the data set should not change observed differences. We randomly permuted 10,000 times the group membership labels between the control stage (E10.5) and E14.5 (Figure 3M). This analysis reveals a significant shift in SSA magnitude at E14.5 compared to earlier stages. These results are in accordance with previously published data using the 3D quantification method (Delaunay et al., 2014).

To ensure that the spindle orientation had no effect on SSA measurement, we plotted the spindle angle deviation against the SSA magnitude. The spindle angle measurement was carried out on dissociated precursors (used in Delaunay et al., 2014) as described in Toyoshima and Nishida (2007). The angle between the axis of the metaphase spindle and the substrate surface was measured using the linear distance (X) and the vertical distance (Y) between the two poles of the metaphase spindles revealed by the pericentrin staining. The calculated angle was denominated α and expressed in degrees. α was measured on a representative sample of metaphase cells at two extreme developmental stages: E10.5+1 DIV (when SSA magnitude is the lowest) and E14.5+1.5 DIV (when the SSA values peak). For each metaphase, the value of α was compared to the value of the 2D SSA. As expected, the spindle deviation was very low and mostly distributed between 0 and 5 degrees at both E10.5+1DIV and E14.5+1.5 DIV. No correlation was observed between α and SSA magnitude at both stages. At E14.5, a stage characterized by high SSA values, the spindle orientations stay close to 5 degrees for most of the population but the number of individuals with greater α increased. However, these individuals did not display a higher SSA. Inversely, at both ages, metaphase displaying a greater angle of deviation exhibits a low level SSA level. For both stages—E10.5 and E14.5—the standard deviations of the spindle angle were respectively 8.14 and 8.5. Altogether, these results formally demonstrate the independence between the spindle deviation and the asymmetry of the mitotic spindle. A deviation from the substrate surface at metaphase will not result in an increase in the measured SSA.

To conclude, the 2D surface area measurements reliably capture SSA distributions as well as efficiently quantifying changes in magnitude during corticogenesis.

### Theoretical relationship between 2D and 3D spindle pole differences: shape independence

To explore the correlation between 2D and 3D quantification, we investigated the theoretical relationship between the 2D and 3D spindle-pole differences Δ and Δ_V_. To do so, we started by modeling the left and right spindle poles as half-spheroids and as right-circular cones having the same axis of revolution. For these two simple models, we found that Δ_V_ is nearly linear in Δ with a slope bounded by 1 and by the ratio of the left-to-right spindle-pole diameter. We then showed that this result holds in the general case where the spindle poles are scaled versions of an arbitrary solid of revolution.

#### Spheroidal and conical models

**Figure 4:**
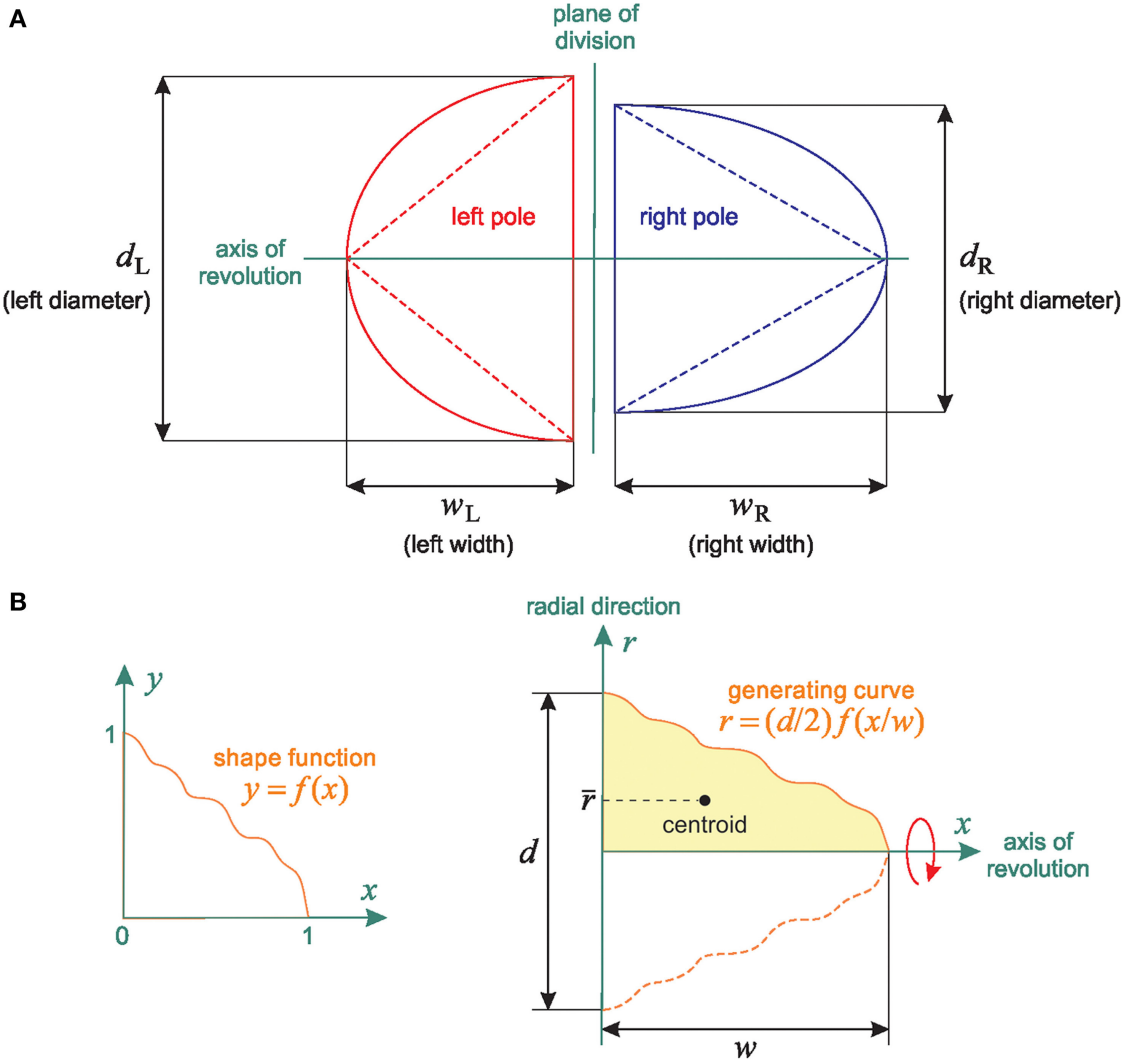
**Linear relationship between 2D and 3D SSA measurements: Shape independence. (A)** Representation of the theoretical spheroidal and conical spindle-pole models: the poles are either half-spheroids or right-circular cones with the same axis of revolution. The spindles are defined by both their base length (*b*_L_ and *b_R_* for the Left and Right spindle pole respectively) and their width (*W*_L_ and *W*_R_, representing the distance between the centrosome and the central spindle). **(B)** General spindle-pole model: a pole with diameter *d* and width *w* is defined by the revolution about the *x*-axis of the region bounded above by the generating curve *r* = (*d*/2)*f*(*x/w*) (The left and right poles have the same shape function *f* but different values of *d* and *w*).

Figure 4A shows a cross-section of these two simple models in a plane containing the axis of revolution. Assume the spindle poles are half-spheroids, and let *P* be a pole with equatorial diameter *d* and polar radius *w*. The volume of *P* is *V* = 2π*d*^2^*w*/3, and the cross-sectional area of *P* (that is, the area of a cross-section of *P* in a plane containing the axis of revolution) is *A* = π *dw*/2. So *V* is proportional to *Ad* (we have *V* = 4*Ad*/3), and hence it follows from the definition of Δ_V_ that

(1) $$\Delta_{\text{V}}\;=\;\frac{\delta A_{\text{L}}-A_{\text{R}}}{\delta A_{\text{L}}+A_{\text{R}}}\times 100\text{ with }\delta \;=\;\frac{d_{\text{L}}}{d_{\text{R}}},$$

where *d*_L_ and *d*_R_ are the diameters of the left and right poles, respectively. The conical model leads to the same result; indeed, a right-circular cone with diameter *d* and height *w* has volume *V* = π *d*^2^*w*/12 and cross-sectional area *A* = *dw*/2, and so *V* is proportional to *Ad*, as in the spheroidal model.

Equation (1) can be rewritten as

(2) $$\;\Delta_{\text{V}}\;=\;\left(\delta \frac{A_{\text{L}}-A_{\text{R}}}{\delta A_{\text{L}}+A_{\text{R}}}+\left(\delta -1\right)\frac{A_{\text{R}}}{\delta A_{\text{L}}+A_{\text{R}}}\right)\times 100$$

(3) $$\text{or }\Delta_{\text{V}}\;=\;\left(\frac{A_{\text{L}}-A_{\text{R}}}{\delta A_{\text{L}}+A_{\text{R}}}+\left(\delta -1\right)\frac{A_{\text{L}}}{\delta A_{\text{L}}+A_{\text{R}}}\right)\times 100.$$

Since *A*_L_ ≥ A_R_, we have

(4) $$\frac{A_{\text{L}}-A_{\text{R}}}{\delta A_{\text{L}}+A_{\text{R}}}\in \left\{ \begin{array}{l} {}[△/\delta ,△]\text{ if }\delta >1\\ {}[△,△/\delta ]\text{ if }\delta <1 \end{array}\right.$$

(5) $$\text{and }\frac{A_{\text{R}}}{\delta A_{\text{L}}+A_{\text{R}}}\leq \frac{1}{\delta +1}\leq \frac{A_{\text{L}}}{\delta A_{\text{L}}+A_{\text{R}}}$$

We deduce from Equations (2)–(5) that

(6) $$\begin{array}{l} \min\left(\delta ,1\right)△+\varepsilon \left(\delta \right)\leq \Delta_{\text{V}}\leq \max\left(\delta ,1\right)△+\varepsilon \left(\delta \right)\\ \text{ with }\varepsilon \left(\delta \right)=\frac{\delta -1}{\delta +1}\times 100. \end{array}$$

In practice, the left-to-right diameter ratio δ is close to one (for example, in our data, the sample mean and standard deviation of δ are 1.09 and 0.12, respectively). Therefore, it follows from Equation (6) that Δ_V_ is a nearly linear function of Δ for both the spheroidal and conical models.

#### General case

The equivalence between the spheroidal and conical models in terms of the relationship between Δ and Δ_V_ motivates a generalization: we now assume that the left and right spindle poles are solids of revolution with generating curves obtained by scaling the value and the argument of an arbitrary function *f*: [0, 1] → ℝ_+_. This general model is schematized in Figure 4B. The left and right spindle poles have the same axis of revolution and their shapes differ only in the values of the diameter *d* and the width *w* (for example, we obtain the spheroidal and conical models by respectively setting $f(x)=\sqrt{1-x^{2}}$ and *f* (*x*) = 1 − *x*). Our only assumptions on the shape function *f* are that it is continuous and such that *f* (0) = 1 and *f* (1) = 0.

Let us temporarily drop the subscripts “L” and “R” for simplicity. Formally, a spindle pole *P* is obtained by rotating the region

(7) $$D=\left\{\left(x,r)\in {\mathbb{R}}_{+}\times {\mathbb{R}}_{+}\;|\;x\leq w\text{ and }r\leq (d/2)f(x/w\right)\right\}$$

about the *x* axis. According to Pappus' centroid theorem, the volume of *P* is

(8) $$V\;=\;2\pi A(D)\bar{r}$$

where *A(D)* denotes the area of *D* and *r* is the distance of the centroid of *D* to the axis of revolution. By definition,

(9) $$A(D)\;=\frac{d}{2}\int_{0}^{w}f(x/w)\text{d}x$$

(10) $$\text{and }\bar{r}=\frac{1}{A(D)}\int_{0}^{w}\left(\int_{0}^{\left(d/2)f(x/w\right)}r\text{d}r\right)\text{d}x,$$

or equivalently,

(11) $$A(D)=\frac{dw}{2}\int_{0}^{1}f\left(u\right)\text{d}u$$

(12) $$\text{and }\bar{r}=\frac{d^{2}w}{8A\left(D\right)}\int_{0}^{1}f^{2}\left(u\right)\text{d}u.$$

Let us now reintroduce the subscripts “L” and “R” to distinguish the left and right poles. From Equation (8), we have *V*_L_ = π *A*_L_*r*_L_ and *V*_*R*_ = π *A*_R_*r*_R_. Substituting these two expressions into the definition of Δ_V_ gives

(13) $$\Delta_{\text{V}}=\left(\frac{\rho A_{\text{L}}-A_{\text{R}}}{\rho A_{\text{L}}+A_{\text{R}}}\right)\times \text{100 with }\rho =\frac{{\bar{r}}_{\text{L}}}{{\bar{r}}_{\text{R}}}.$$

Furthermore, by Equation (11), *A*_R_/*A*_L_ = *d*_R_*w*_R_/(*d*_L_*w*_L_), and so it follows from Equation (12) that

(14) $$\rho ={\left(\frac{d_{\text{L}}}{d_{\text{R}}}\right)}^{2}\frac{w_{\text{L}}A_{\text{R}}}{w_{\text{R}}A_{\text{L}}}=\frac{d_{\text{L}}}{d_{\text{R}}}=\delta .$$

In other words, Equation (1) holds when the spindle poles are solids of revolution defined by an arbitrary shape function. Consequently, the bounds given in Equation (6) remain valid in the general case, and so we conclude that 2D and 3D measurements have the same discriminative ability for SSA assessment.

### Experimental validation of the quasi-linear relationship between 3D and 2D SSA

**Figure 5:**
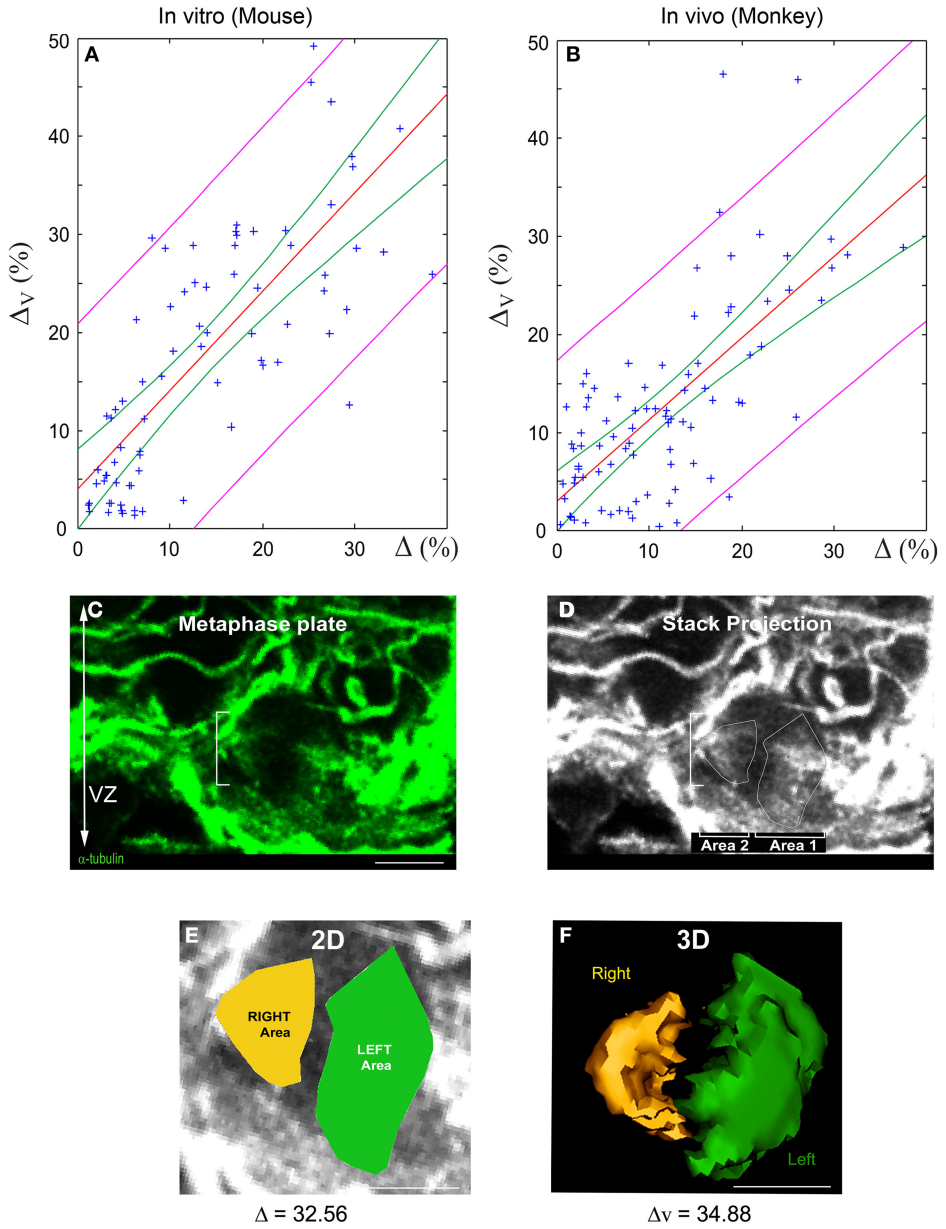
**Linear relationship between 2D and 3D SSA measurements: Experimental validation. (A,B)** Linear regression of 3D vs. 2D SSA measurements. The SSA has been quantified using both 2D and 3D methods and their relationship evaluated for two experimental samples: Mouse AP dividing cells *in vitro* and E63 to E80 Monkey VZ progenitors *in vivo*. **(A)** 2D vs. 3D SSA quantification comparison for *in vitro* mouse. **(B)** 2D vs. 3D SSA quantification comparison for Monkey *in vivo*. The regression line is displayed in red, the magenta curves delimit the 95% pointwise confidence band, and the green curves delimit the 95% Working-Hotelling confidence band. **(C–F)** 2D and 3D SSA in the Monkey VZ. **(C)** Optical sections of an E80 Monkey VZ stained with α-tubulin. The white dashes delimit a cell in metaphase. **(D)** Manually drawn area 1 and 2 on the maximal stack projection. **(E)** 2D SSA area determination. The difference between the Left (green) and the Right area (yellow) is expressed in percentage and is greater than 10, typical of an asymmetric cell. **(F)** 3D volume rendering and calculation of the same cells. Note the proximity between 2D and 3D values. Scale bars: 10 μm.

We performed linear regression analyses to validate the quasi-linear relationship (Equation 6) between 3D and 2D spindle-pole differences. Two separate samples were analyzed: (i) dissociated cortical progenitors (from E10 to E16, Figure 3) and (ii) monkey VZ precursors *in situ* (from E63 to E80, Figures 5A–F). The results are summarized in Figure 5. The green and magenta curves respectively delimit the 95% simultaneous and pointwise confidence bands; that is, the true regression lines lie between the green curves with a probability of 95%, and given a 2D measurement Δ^*^, there is a 95% probability that the corresponding 3D measurement is bounded by the magenta curves at Δ = Δ^*^. In accordance with the bounds given in Equation (6), the slopes of the regression lines are close to one: the regression line L1 of the *in vitro* mouse data has a slope of 1.009 with a standard deviation of 0.095, and the regression line L2 of the *in vivo* monkey data has a slope of 0.831 with a standard deviation of 0.084 (the intercepts of L1 and L2 are smaller than 4%). The 95% confidence intervals for the true slopes of L1 and L2 are (0.82, 1.20) and (0.66, 1.00), respectively. That is, we estimate with 95% confidence that if the 2D spindle-pole difference increases by 10%, then the mean 3D spindle-pole difference increases by somewhere between 8.2 and 12% in the case of the *in vitro* mouse data, and between 6.6 and 10% in the case of the *in situ* monkey data—this further confirms the proportionality between 2D and 3D measurements, and hence their equivalent discriminative power.

## Discussion

In the present study, we provide two distinct methods to achieve accurate SSA measurements—3D volume determination and 2D area measurement. Theoretical and empirical comparisons of the two methods show a nearly linear relationship. Using Pappus'centroid theorem, we demonstrated that this relationship is independent of the spindle shape. This structural property rules out any potential bias of spindle deformation on SSA determination, thereby further supporting the validity of SSA 2D measurement. Finally, we confirmed our theoretical findings by performing linear regression analyses on *in vitro* (mouse) and *in situ* (monkey) metaphase cell samples.

### Methodological considerations

3D volume measurement requires the analysis of spindle contours on approximately ten individual optical sections. Therefore, minute errors in delineating the pixel contours (2D) are amplified when summing the results. By contrast, errors in spindle contour delineation will have a smaller impact on 2D surface measurement, where the whole spindle apparatus is reduced to a single plane. These technical discrepancies could explain the minute variations in linearity observed experimentally when comparing 2D and 3D SSA measurements (Figure 5).

For both methods, we selectively sampled the metaphase cell populations. Only cells displaying equal sized centrosomes were taken into consideration, a configuration that favors cells harboring a spindle aligned parallel to the acquisition plane. *In vitro*, the spindle apparatus is easily accessible and cells mostly divide parallel to the coated surface—our observations show that 86% (E10) and 82% (E14) of precursors exhibit a spindle angle deviation which ranges between 0 and 5 degrees. When we compared the spindle angle deviation with the SSA magnitude, we found no correlation between those two parameters, demonstrating the independence between the spindle angle deviation and the spindle size asymmetry (SSA). *In situ* however, cells could potentially divide along all axis, causing a bias in the representation of rostro-caudally dividing cells. In the neuroepithelium, apical progenitor have been described as aligning along the planar axis before rotating along the rostro-caudal axis during metaphase (Peyre et al., 2011). Such a rotation pattern has also been observed under live imaging in mouse embryonic cortex, in dividing apical progenitors expressing alpha-tubulinEGFP at metaphase (Delaunay et al., 2014). The spindles were aligning along the planar axis, docking, rotating around the caudo-rostral axis, coming back to their original planar position, moving around the planar axis or staying at the same place, rotating again along the rostro-caudal axis and so on until the beginning of anaphase. We quantified the 2D and 3D spindle pole differences for each sequential moment of planary aligned spindles–between the rounds of rostro-caudal rotations and observed that the spindle pole size difference was stable (Delaunay et al., 2014; Figure 1). In all cases, asymmetric spindle are observed to remain asymmetric and a symmetric spindle remains symmetric, independently of the rostro-caudal rotations. Therefore, population sampling is unlikely to affect SSA measurements. This is further supported by the fact that SSA can be observed at similar frequencies on coronal (mouse) (Delaunay et al., 2014) and parasagittal (monkey) sections. Thus, SSA can be unambiguously determined in 2D. Taken together, our results provide a reliable method for SSA quantification in cortical apical progenitors, a method that can be extended to other cell types.

### 2D vs. 3D SSA quantification

Alongside SSA, changes in plane of division orientation have always been considered as major determinants of ACD in the cerebral cortex. Spindle orientation—although controversial— could regulate the fate of cortical progenitors by controlling the balance between proliferation and differentiation (Chenn and McConnell, 1995; Yingling et al., 2008; Godin et al., 2010). Generally quantified in 2D, spindle orientation varies between two extremes: horizontal divisions (0–15° angle, relative to the referential axis) or vertical divisions (75–90°). Horizontal divisions are associated with symmetric divisions and vertical divisions with asymmetric divisions. A recent work from the Knoblich group reports a new method for 3D analysis of the mitotic plane orientation (Juschke et al., 2014). The authors approximate the mitotic cell by a sphere and mathematically define the spindle orientation by elongating the spindle axis so that it interacts with the surface of the sphere. Under these conditions, randomness results in a predominance of horizontally oriented spindles (close to 50%), a result which could be explained by true stochasticity. To refine their 3D analysis, the authors have introduced two novel parameters: λ h and λ v, respectively the horizontal and vertical enrichment. This method excludes the effects of planar cell polarity (important in numerous epithelia) and assumes symmetry around the z axis. Juschke et al used this method to assess the role of two proteins: PP4C and mInsc on the plane of division orientation. Interestingly, they report an equivalence between 2D and 3D results for the PP4C-KO with no distinction between randomization or horizontal vs. vertical enrichment. The same analysis performed with the mInsc protein reveals a horizontal enrichment in the KO and a vertical enrichment in mInsc overexpressing cells. Of note, the instructive effect of mInsc overexpression on vertical divisions has been reported by another group, using 2D analysis (Konno et al., 2008; Figure 3). The congruence between 2D and 3D spindle orientation analysis argues in favor of the robustness of the 2D SSA assessment methodology.

SSA has recently been documented in the developing mouse cortex, highlighting its importance in Vertebrates and Invertebrates ACD regulation (Delaunay et al., 2014). Previously our evidence for a role of SSA in ACD was in rodents. The present data provide the first evidence of SSA in primate apical progenitors. This indicates that despite the difference in the basic cellular (Betizeau et al., 2013) and molecular regulation (Arcila et al., 2014) between rodent and primate corticogenesis, SSA operates in similar fashion in both orders. Likely, SSA will play a crucial role in controlling ACD in the VZ but also in the OSVZ in the primate lineage. This maintenance of SSA in asymmetrically dividing progenitors argues in favor of its crucial importance during cortical development, thus, highlighting the need for an accurate yet methodically simple quantification process such as the one proposed here.

## Author contributions

Delphine Delaunay and Colette Dehay conceived and designed experiments. Delphine Delaunay performed experiments and analyzed the data. Marc C. Robini performed the mathematical and statistical analysis. All authors wrote the paper.

### Conflict of interest statement

The authors declare that the research was conducted in the absence of any commercial or financial relationships that could be construed as a potential conflict of interest.

## Supplementary material

The VolumeJ program,the “utilFuncs.R” script and the related instructions are provided as text format.
