# Supplementary material for: Mitotic spindle asymmetry in rodents and primates: 2D vs. 3D measurement methodologies
Source: Front Cell Neurosci. 2015 Feb 9;9:33. doi: 10.3389/fncel.2015.00033 (PMC4321609; doi:10.3389/fncel.2015.00033)
Supplement: Supplementary file 1 [file DataSheet1.PDF]

volume quanti 1_0.pdf

File Edit Font Macros Debug

```
//////////////////////////////////////////////////////////
/*Cette macro aide l'utilisateur à séparer deux structures de faisceaux de chromatine, quantifie leur volume et propose une représentation 3D surfacique.

Date : 12/06/13
Auteur : Denis Ressnikoff
version : 1.0

mise a jour :

contact:
denis.ressnikoff@univ-lyon1.fr
Centre Commun de Quantimétrie
http://quantimetrie.univ-lyon1.fr/

*/

//inistialisation
run("Clear Results");
run("Select None");
run("Colors...", "foreground=white background=black selection=red");
im=getTitle();
imDir=getInfo("image.directory");
getVoxelSize(pWidth, pHeight, depth, unit);


 if (isOpen("Results")) {
       selectWindow("Results");
       run("Close" );
}

 if (isOpen("ROI Manager")) {
       selectWindow("ROI Manager");
       run("Close" );
}

//extration manuelle de la zone d'intérêt
setTool("polygon");
waitForUser("Extraction", "Sélectionnez la zone d'intêret au plus proche et validez ici");
run("Clear Outside", "stack");
run("Crop");
run("Select None");
run("Set... ", "zoom=400");
saveAs("Tiff",imDir+im+"_ROI");

//Création d'une image clone pour le masque binaire
run("Duplicate...", "title="+im+"_mask"+" duplicate range=1-"+nSlices);
run("Set... ", "zoom=400");
run("Tile");
```

volume quanti 1_0.pdf

File Edit Font Macros Debug

```
//Seuillage manuel
run("Threshold...");
setAutoThreshold("IJ_IsoData dark");
setOption("BlackBackground", true);
waitForUser("Seuillage", "Veuillez ajuster le seuillage et valider ici");
selectWindow(im+"_mask");
run("Convert to Mask", "method=IJ_IsoData background=Dark black");
selectWindow("Threshold");
run("Close");

//fermeture, mise en forme du masque binaire
run("Options...", "iterations=1 count=1 black pad edm=Overwrite do=Close stack");

//séparation manuelle des deux structures
setTool("wand");
waitForUser("séparation des structures", "Veuillez selectionner une structure sur tous les plans \n (touche T pour valider, touche shift pour aditionner 2 sélections) \n puis valider ici")
getDimensions(width, height, channels, slices, frames);
newImage(im+"_mask_2", "8-bit Black ",width, height, slices);
setVoxelSize(pWidth, pHeight, depth, unit);
run("Set... ", "zoom=400");
run("Tile");
nbRoi=roiManager("count");

//copie dans 2 piles distinctes
for (i=0;i<nbRoi;i++) {
	selectWindow(im+"_mask");
	roiManager("Select", i);
	run("Clear", "slice");
	selectWindow(im+"_mask_2");
	roiManager("Select", i);
	run("Fill", "slice");
}

//ajout des images vides au début et à la fin pour fermer les volumes.
selectWindow(im+"_mask");
run("Select None");
setSlice(slices);
run("Add Slice");
setSlice(1);
run("Cut");
run("Add Slice");
setSlice(2);
run("Paste");


selectWindow(im+"_mask_2");
run("Select None");
setSlice(slices);
run("Add Slice");
setSlice(1);
run("Cut");
run("Add Slice");
setSlice(2);
run("Paste");
```

```
//quantification 3D
run("Set 3D Measurements", "volume nb_of_obj._voxels dots_size=5 font_size=10 store_results_within_a_table_named_after_the_image_(macro_friendly) redirect_to=none");
selectWindow(im+"_mask_2");
run("3D object counter...", "threshold=128 slice=4 min.=0 max.=100000000 statistics");

selectWindow(im+"_mask");
run("3D object counter...", "threshold=128 slice=4 min.=0 max.=100000000 statistics");

//représentation 3D
run("3D Viewer");
call("ij3d.ImageJ3DViewer.setCoordinateSystem", "false");
call("ij3d.ImageJ3DViewer.add", im+"_mask", "Red", im+"_mask", "50", "true", "true", "true", "2", "2");
call("ij3d.ImageJ3DViewer.add", im+"_mask_2", "Green", im+"_mask_2", "50", "true", "true", "true", "2", "2");

run("Merge Channels...", "c1="+im+"_mask c2="+im+"_mask_2 keep");
rename(im+"_color_Mask");
saveAs("Tiff",imDir+im+"_color_Mask");
run("Set... ", "zoom=400");

//enregistrement des résultats
selectWindow(im+"_mask");
saveAs("Tiff",imDir+im+"_Mask_1");
close();
selectWindow(im+"_mask_2");
saveAs("Tiff",imDir+im+"_Mask_2");
close();

selectWindow("Statistics for "+im+"_mask");
saveAs("Results",imDir+im+"_Mask_1.xls");

selectWindow("Statistics for "+im+"_mask_2");
saveAs("Results", imDir+im+"_Mask_2.xls");
```

utilFuncs.R

```
### read-in data
###  data format:  left right Cond (day/construct/etc.)
read.data <- function(f, control.lev = NULL, sep = ";", ...){
	prts <- strsplit(f, "\\.")[[1]]
	if (prts[length(prts)] != "csv")
		stop("File must be in csv format")
	d <- read.csv(f, sep = sep, ...)
	if (any(d$left < d$right))
	   d[which(d$left < d$right), ] <- d[which(d$left < d$right), c(2,
1, 3)]
	d[[3]] <- if(!is.null(control.lev)) relevel(d[[3]], control.lev)
else d[[3]]
	d$LmR <- with(d, left - right)
	d
}

### permtest

perm.test <- function(d, N = 10,  mod){
	mod <- if (missing(mod)) {
		iv <- names(d)[3]
		f <- substitute(as.formula(LmR ~ V), list(V = as.name(iv)))
		f <- as.formula(f)
		lm(f, d)
	} else mod
	nlevs <- length(levels(d[[3]]))
	pt.mat <- sapply(seq(N), function(x, d, mod){
		nr <- nrow(d)
		d[[3]] <- sample(d[[3]], nr)
		coef(update(mod, data = d))[seq(nlevs)[-1]]
	}, d = d, mod = mod)
	if (is.null(dim(pt.mat))) dim(pt.mat) <- c(1, length(pt.mat))
## need to make this general for n columns
	cf <- coef(mod)[-1]
	pt.mat <- cbind(pt.mat, cf)
	pvals_1s <- sapply(seq(length(cf)), function(ix) sum(pt.mat[ix, ]
>= cf[ix])/(N + 1))
	pvals_2s <- sapply(seq(length(cf)), function(ix) sum(abs(pt.mat[ix,
]) >= abs(cf[ix]))/(N + 1))
	res <- list(res = pt.mat, p = rbind(pvals_1s, pvals_2s), refCoefs =
cf)
	class(res) <- 'prm.test'
	res
}

med.perm.test <- function(d, N = 10){

	meds <- tapply(d$LmR, d[3], median)
	dmeds <- (meds - meds[1])[-1]
	nlevs <- length(levels(d[[3]]))
	pt.mat <- sapply(seq(N), function(x, d){
		nr <- nrow(d)
		d[[3]] <- sample(d[[3]], nr)
		meds <- tapply(d$LmR, d[3], median)
		dmeds <- (meds - meds[1])[-1]
```

```
      }, d = d)
      if (is.null(dim(pt.mat))) dim(pt.mat) <- c(1, length(pt.mat))
## need to make this general for n columns
      cf <- dmeds
      pt.mat <- cbind(pt.mat, cf)
      pvals_1s <- sapply(seq(length(cf)), function(ix) sum(pt.mat[ix, ]
>= cf[ix])/(N + 1))
      pvals_2s <- sapply(seq(length(cf)), function(ix) sum(abs(pt.mat[ix,
]) >= abs(cf[ix]))/(N + 1))
      res <- list(res = pt.mat, p = rbind(pvals_1s, pvals_2s), refCoefs =
cf)
      class(res) <- 'prm.test'
      res
}

fun.perm.test <- function(d, N = 10, fun = median){
      meds <- tapply(d$LmR, d[3], fun)
      dmeds <- (meds - meds[1])[-1]
      nlevs <- length(levels(d[[3]]))
      pt.mat <- sapply(seq(N), function(x, d){
            nr <- nrow(d)
            d[[3]] <- sample(d[[3]], nr)
            meds <- tapply(d$LmR, d[3], fun)
            dmeds <- (meds - meds[1])[-1]
      }, d = d)
      if (is.null(dim(pt.mat))) dim(pt.mat) <- c(1, length(pt.mat))
## need to make this general for n columns
      cf <- dmeds
      pt.mat <- cbind(pt.mat, cf)
      pvals_1s <- sapply(seq(length(cf)), function(ix) sum(pt.mat[ix, ]
>= cf[ix])/(N + 1))
      pvals_2s <- sapply(seq(length(cf)), function(ix) sum(abs(pt.mat[ix,
]) >= abs(cf[ix]))/(N + 1))
      res <- list(res = pt.mat, p = rbind(pvals_1s, pvals_2s), refCoefs =
cf)
      class(res) <- 'prm.test'
      res
}


### plot permtest
plot.prm.test <- function(x, tpos = c(-3, 200), layout =
n2mfrow(length(x$p[1, ])),
      twosided = TRUE, signif.digits = 3, ...){
      opar <- par(mfrow = layout)
      on.exit(par(opar))
      for(ix in seq(length(x$p[twosided + 1, ]))){
            hist(x$res[ix, ], main = names(x$refCoefs)[ix], ...)
            abline(v = x$refCoefs[ix], lwd = 3)
            text(tpos[1], tpos[2], paste("p =", round(x$p[twosided + 1,
ix], signif.digits)), adj = 0)
      }
}

### folded-normal test
fnorm.test <- function(data, ...){
      require(VGAM)
      iv <- names(data)[3]
```

```
	f <- substitute(as.formula(LmR ~ V), list(V = as.name(iv)))
	f <- as.formula(f)
	mod <- vglm(f, fnormal1, data = data, ...)
	print(summary(mod))
	invisible(mod)
}

### plot folded normal results
plotPred <- function(mod, d, layout = n2mfrow(length(coef(mod))/2),...){
	require(VGAM)
	if (!inherits(mod, "vglm")) stop("Object must be of class vglm!")
	cf <- coef(mod)
	MN <- cf[seq(1, length(cf), 2)]
	SD <- cf[seq(2, length(cf), 2)]
	MN <- MN[1] + c(0, MN[-1])
	SD <- exp(SD[1] + c(0, SD[-1]))
	names(MN) <- names(SD) <- levels(d[[3]])
	opar <- par(mfrow = layout)
	on.exit(par(opar))
	for(ix in levels( d[[3]] )){
		tmpd <- droplevels(subset(d, get(names(d)[3]) == ix))
		hist(tmpd$LmR, freq = FALSE,
			main = ix, ...)
		xx <- seq(0, max(d$LmR))
		lines(xx, dfnorm(xx, MN[ix], SD[ix]))
	}
}
```

1 A set of utilities for analyzing differences in spindle size

K. Knoblauch July 27, 2012

I have written a set of functions that should, in principle, make it relatively easy to analyze the differences in measured spindle size. The functions are all in one file named "utilFuncs.R". The functions become accessible by reading them into memory in R using the `source` function, as will be illustrated below.

There are currently 5 functions coded. These are

1. `read.data` will read in the data from a file in csv format, if in the correct format, into a data frame in memory, as described below.

2. `perm.test` will perform a permutation test on the data set. It returns an object of class "prm.test" for which a plot method has been defined.

3. `plot.prm.test` is a plot method defined for objects of class "prm.test".

4. `fnorm.test` will fit a linear model following a folded-normal distribution to the data set.

5. `plotPred` will plot the density histogram of the data and the folded-normal predictions from the previous test.

1.1 Loading the functions in memory

To load the functions in memory, you need to have the file "utilFuncs.R" in your working directory. You need to start R and change the working directory (see the menu item) to the one that contains this file. After having done this, you can source its contents into memory with the following command.

```
source("utilFuncs.R")
```

The most likely errors are that the file name was mispelled (case matters) or that the file was not in the working directory. You can check that it is there by typing

```
dir()

## [1] "Asym.csv"          "Constructs.csv"   "Instructions.Rnw"
## [4] "Instructions.pdf" "Instructions.tex" "Sweavel.sty"
## [7] "cache"             "figure"           "utilFuncs.R"
```

which displays the files in the working directory as shown above for my working directory.

If the `source` command works, the five functions should have been loaded in your workspace. This can be checked by using the `ls` command that lists the objects in the current workspace as follows

```
ls()

## [1] "fnorm.test"    "perm.test"     "plot.prm.test" "plotPred"
## [5] "read.data"
```

1.2 Data format

Before reading the data into memory, we describe the expected format of the data file. First, the file should be in csv (comma separated value) format. This format can be obtained by saving the file from an Excel spreadsheet and specifying it to be of this format. It will have the extension ".csv" as shown on two of the files in the directory listing above.

The file is expected to have 3 columns with headers "left", "right" and a third column giving the name of the condition. For example, the third column is named "Day" in the file "Asym.csv" and it is named "Construct" in the file "Constructs.csv". The first two columns give the measurements of the left and right spindles and the third specifies the condition under which the measurement was made. For example, the measurements for the file "Asym.csv" are labelled with the conditions "e10", "e11.5" and "e14.5". These will correspond to factor levels in the models fit to the data. For the file "Constructs.csv", there are four different levels.

The measurments from all of the levels should be in the same file. There is one row per cell with three pieces of data (columns) per row (cell) as described above.

1.3 Reading in the data

The data is loaded into the workspace using the function `read.data`. It minimally takes the quoted file name containing the data set. It can optionally take a second argument, `control.lev`, that specifies which level of the condition variable (Day or Construct, in the examples, above) should be considered the control level. By default, the levels are specified in alphabetical order with the first one being the control level. This option allows one to alter this situation.

The data set in the "Asym.csv" file is read into memory as follows.

```
Day.df <- read.data("Asym.csv")
```

The function returns a data frame. Unless it is assigned to a variable, as above, it will simply be printed out in the console window. Above, we assigned it to a variable named `Asym.df`. I use the extension `.df` to remind myself that it is a data frame, but any legal name can be used. The assignment operator is the arrow created with two keystrokes, the less than symbol, '<' followed by the minus sign '−', and which gives '<−'. It becomes second nature with practice.

Note that we did not specify the optional second option above We could have also written

```
Day.df <- read.data("Asym.csv", control.lev = "e10")
```

but since "e10" is alphabetically first, it was not necessary. Note that we use '=' when specifying function arguments and not '<−'.

Just to show the use of the `control.lev` option in a situation where it makes a difference, consider the data set in the file "Constructs.csv". We will load it two different ways, but instead of assigning it to a variable, we will make it the argument of the `str` function so that we can see the structure of the output of the `read.data` function.

```
str(
  read.data("Constructs.csv")
  )

## 'data.frame': 91 obs. of  4 variables:
##  $ left     : num  68.5 54 55.1 51.7 75.4 ...
##  $ right    : num  31.5 46 44.9 48.3 24.6 ...
```

```
##  $ Construct: Factor w/ 4 levels "fz","pegfp","vgl2egfp",..: 1 1 1 1 1 1 1 1 1 1 ...
##  $ LmR      : num  36.94 7.94 10.12 3.32 50.84 ...

str(
  read.data("Constructs.csv", control.lev = "pegfp")
  )

## 'data.frame': 91 obs. of  4 variables:
##  $ left     : num  68.5 54 55.1 51.7 75.4 ...
##  $ right    : num  31.5 46 44.9 48.3 24.6 ...
##  $ Construct: Factor w/ 4 levels "pegfp","fz","vgl2egfp",..: 2 2 2 2 2 2 2 2 2 2 ...
##  $ LmR      : num  36.94 7.94 10.12 3.32 50.84 ...
```

In the second case, we assigned the level "pegfp" to be the control level instead of the first level in alphabetical order, "fz" as in the first case. Note the difference in order of how the levels are specified in the `Construct` component of the data sets that were read-in with the two versions of the command above.

The `read.data` function does several other things besides just reading-in the data. It first checks that the extension of the file is, in fact, "csv" and if it is not, it aborts with an error message. After successfully reading in the file, it checks if any of the left values are less than the corresponding right values, and if so, it reverses them. Then, if the `control.lev` argument is set, it relevels the condition factor so that the specified level is the first level. Then, it adds a new column, `LmR`, that is the difference between the left and right values. It is this value that will be analyzed in successive functions. Finally, it returns the data frame, which is why it needs to be assigned to a variable.

If the steps above ran successfully, i.e., no error messages were generated, then an object named `Day.df` should be in the workspace. Recall we examine what is in the workspace with the function `ls` (think list).

```
ls()

## [1] "Day.df"         "fnorm.test"     "perm.test"      "plot.prm.test"
## [5] "plotPred"       "read.data"
```

Note the object `Day.df` in the workspace now. To verify its structure, we use the `str` command.

```
str(Day.df)

## 'data.frame': 288 obs. of  4 variables:
##  $ left : num  53.5 53.2 50.8 52 51.1 ...
##  $ right: num  46.5 46.8 49.2 48 48.9 ...
##  $ Day  : Factor w/ 3 levels "e10","e11.5",..: 1 1 1 1 1 1 1 1 1 1 ...
##  $ LmR  : num  7.01 6.5 1.64 4.04 2.13 ...
```

which shows that `Day.df` is a data frame with 288 observations (rows) and 4 variables (columns). The variables are left, right, Day and LmR. The first two are numeric. The third is a factor with 3 levels and the first level is "e10" and the fourth is also numeric.

1.3.1 Brief summary of a few basic housekeeping functions

It is perhaps useful to review the basic R commands that we have used here.

1. `dir` shows what files are in the working directory.

2. `ls` shows what objects are in the workspace

3. `source` reads a file of R commands into memory and executes them. Its use above was to simply read-in function definitions.

4. `str` is used to examine the structure of a data object.

1.4 Permutation test

The function `perm.test` performs a permutation test on the differences of the variable that codes the difference of left and right values, `LmR`, from the first level of the conditon variable. That is, under the hypothesis that the asymmetry (left - right difference) is the same for all condition levels, we should expect that the mean of each level should not differ from that of the control level on average if we assign each cell to a random level. The permutation test makes such assignments a large number of times to generate the distribution of differences under this hypothesis. The proportion of cases found to be as extreme or more extreme than the observed values gives the probability of the observed differences under the null hypothesis.

The function requires minimally two arguments, the data frame to be tested and `N` the number of permutations to run. This should normally be a large number such as 10000.

```
Day.pt <- perm.test(Day.df, N = 10000)
```

The function returns an object of class "prm.test" and we have assigned its output to the variable `Day.pt`. Here, again, I gave the variable an extension to aid in remembering what it contains, but the naming is arbitrary. An object of this class is a list that contains 3 components, a matrix giving the results of the `N` permutaton test with one additional element for the observed data, the p-values for the comparisons of the first level with each of the others, and observed differences from the original data, i.e., how much does each condition differ from the first level. We examine its structure with the `str` function.

```
str(Day.pt)

## List of 3
##  $ res      : num [1:2, 1:10001] 1.643 0.955 -0.567 -0.101 0.106 ...
##   ..- attr(*, "dimnames")=List of 2
##   .. ..$ : chr [1:2] "Daye11.5" "Daye14.5"
##   .. ..$ : chr [1:10001] "" "" "" "" ...
##  $ p        : num [1:2] 0.2757 0.0001
##  $ refCoefs: Named num [1:2] 0.591 4.053
##   ..- attr(*, "names")= chr [1:2] "Daye11.5" "Daye14.5"
##  - attr(*, "class")= chr "prm.test"
```

In general, our main interest is in obtaining the p-values and perhaps visualizing the permutation distributions. These are obtained with the plot method. We note three additional arguments specified, first of which is explained below. The `layout` argument can be used to control the layout of the plots, here 1 by 2 and the `signif.digits` argument controls the rounding of the p-values included in the plots.

```
plot(Day.pt, tpos = c(-3.5, 1400), layout = c(1, 2), signif.digits = 4)
```

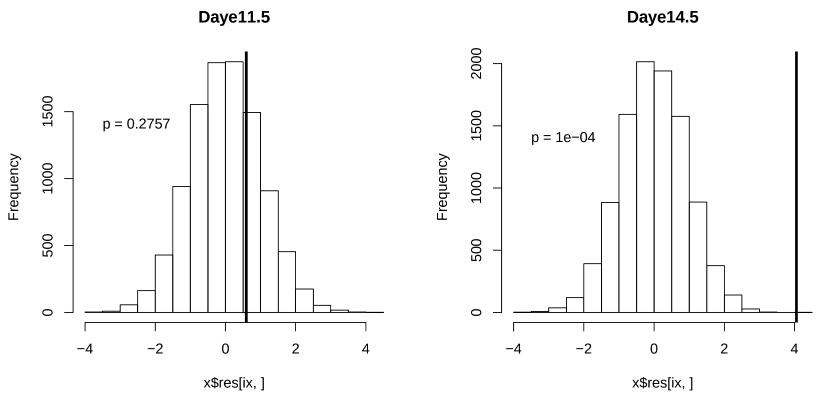

For this data set, there are three levels of the variable Day, so there are two comparisons, the second and third days with the first. Each histogram shows the 10000 permutations. The vertical bar indicates the difference in mean of that day from the control in the actual, unpermuted data set. The p-values are indicated in the upper left of each graph, i.e., the proportion of cases as extreme or more extreme than the observed value. To position the p-value, the plot function can take a second argument `tpos` that indicates a vector (created with the `c` function) that indicates the x and y positions to plot the p-value.

Note that even though the function that we defined was named `plot.prm.test`, it was only necessary to use `plot`. This is because the object returned by the `perm.test` function is of class "prm.test" and so when given to the generic `plot` function, it knows which plot method to call, i.e., the one appropriate to this class of object that we defined and read into memory at the beginning with the `source` function.

The `plot` function can take additional arguments to specify names for the axes, different limits to the axes, etc.

1.5 Fitting the folded-normal distribution

Because the designations of left and right in the spindle data are arbitrary, the left is arbitrarily assigned the larger value. This is equivalent to measuring the absolute value or the magnitude of the difference between the two sides. If the difference is normally distributed then the magntude follows a folded-normal distribution.

The folded normal distribution can be fit with the `vglm` function from the **VGAM** package using the `fnormal1` link. To simplify things, I wrote a wrapper function around this funtion, `fnorm.test`, that performs a simple test of the differences in the means and standard deviations between the conditon levels.

The function takes a data frame as its argument and prints out a summary of the results, but returns an object that can be probed with other methods, if necessary.

```
suppressWarnings(Day.fnt <- fnorm.test(Day.df))

##
## Call:
## vglm(formula = f, family = fnormal1, data = data)
```

```
##
## Coefficients:
##                Estimate Std. Error z value
## (Intercept):1     -0.22      4.058  -0.054
## (Intercept):2      1.92      0.078  24.740
## Daye11.5:1         0.16      6.238   0.026
## Daye11.5:2         0.12      0.110   1.113
## Daye14.5:1        -0.45      7.583  -0.059
## Daye14.5:2         0.59      0.107   5.518
##
## Number of linear predictors:  2
##
## Names of linear predictors: mean, log(sd)
##
## Dispersion Parameter for fnormal1 family:   1
##
## Log-likelihood: -840.8 on 570 degrees of freedom
##
## Number of iterations: 13
```

The summary shows the estimates from fitting the folded-normal model to the data with their estimated standard errors and the ratio of the mean and standard error giving a z-value. No p-value is given but the z-value can be referred to a standard normal distribution to give a sense of how likely the observed differences are. For the model fit to this data set, there are 6 estimates. The first two rows of the summary table give the estimated mean and $\log_e$ SD, respectively, for the control case, “e10” here. The z-value does not provide evidence that the control mean differs from zero, i.e., the left and right differences are symmetric, but the SD is quite different from a value of 1 (log SD = 0). The SD of the control distribution would be obtained by taking the anti-log of the estimated value, exp(1.923) = 6.84.

The next four rows give means and log(SD) for the differences of the values estimated at days 11.5 and 14.5 with respect to the control. If any of these are significant, it indicates a significant difference from the control level. Examining the z-values only suggests that the SD of day 14.5 differs significantly from that of the control, with the other values not very different. The factor by which the SD of day 14.5 is estimated to be greater than the control is obtained by taking the anti-log of the estimate: exp(0.588) = 1.8, or almost twice as great.

One way to evaluate if the model is a good fit is to plot the distribution of the data with the distribution given by estimated parameters plotted over it. We do that with the `plotPred` function. It minimally takes 2 arguments, a model object that has been returned by running `fnorm.test`, as above, and the data frame withe the data. Additional graphic parameters can be added as arguments to make the plots more presentable, as shown below with the parameters `xlim` and `ylim` that set the limits of the x- and y-axes, respectively, to be the same for all of the plots, for easing comparisions and the `breaks` parameter that sets the bins for use in the histogram. Note that the histogram values have been normalized by the total number of data points so that the ordinate is labelled as “Density” rather than “Frequency”. Other parameters can be added to adjust the appearance of the graph.

```
plotPred(Day.fnt, Day.df, xlim = c(0, 50),
        breaks = seq(0, 50, 2.5), ylim = c(0, 0.15))
```

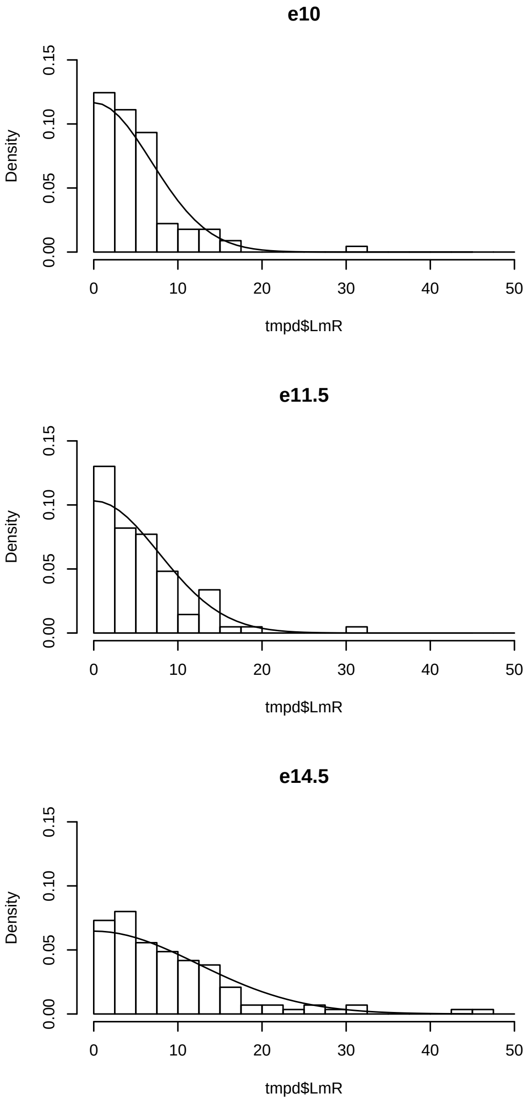

The solid lines correspond to the best fit folded-normal distribution for each condition.
